# Association of Second-generation Antiandrogens With Depression Among Patients With Prostate Cancer

**DOI:** 10.1001/jamanetworkopen.2021.40803

**Published:** 2021-12-23

**Authors:** Malgorzata K. Nowakowska, Xiudong Lei, Mackenzie R. Wehner, Paul G. Corn, Sharon H. Giordano, Kevin T. Nead

**Affiliations:** 1 School of Medicine, Baylor College of Medicine, Houston, Texas; 2 Department of Health Services Research, University of Texas MD Anderson Cancer Center, Houston; 3 Department of Dermatology, University of Texas MD Anderson Cancer Center, Houston; 4 Department of Genitourinary Medical Oncology, University of Texas MD Anderson Cancer Center, Houston; 5 Department of Breast Medical Oncology, University of Texas MD Anderson Cancer Center, Houston; 6 Department of Radiation Oncology, University of Texas MD Anderson Cancer Center, Houston; 7 Department of Epidemiology, University of Texas MD Anderson Cancer Center, Houston

## Abstract

**Importance:**

Previous studies have shown a consistent association between hormone therapy (HT), such as androgen deprivation therapy, to treat prostate cancer and depression risk. However, the association between second-generation antiandrogens (AAs) and depression is unknown.

**Objective:**

To test the a priori hypothesis that second-generation AAs are associated with an increased risk of depression, including compared with traditional forms of HT.

**Design, Setting, and Participants:**

This retrospective cohort study analyzed patients aged 66 years and older who were diagnosed with prostate cancer without a second cancer in 12 months from January 2011 to December 2015. Patients with continuous Medicare Parts A, B, and D coverage were included. Individuals who received any form of HT prior to prostate cancer diagnosis and those previously diagnosed with depression were excluded. Data were collected from the Surveillance, Epidemiology, and End Results–Medicare and Texas Cancer Registry–Medicare linked databases. Data were analyzed from February to May 2021.

**Exposures:**

The following treatment groups were compared: (1) no HT group, (2) traditional HT group (HT without second-generation AA exposure), and (3) second-generation AA group.

**Main Outcomes and Measures:**

Risk of depression in the second-generation AA group compared with the no HT and traditional HT groups, determined prior to data collection, stratified by diagnosis stage.

**Results:**

Of 210 804 patients diagnosed with prostate cancer during the study window, 30 069 men (11 484 [38%] aged 66-70 years; 22 594 [75%] White) who met inclusion criteria were identified. Overall, 17 710 (59%) received no HT, 11 311 (38%) received traditional HT only, and 1048 (3%) received a second-generation AA. Those receiving a second-generation AA were more likely to be older (aged ≥81 years: second-generation AA group, 246 [24%]; traditional HT group, 1997 [18%]; no HT group, 1173 [7%]) and present with advanced disease (eg, distant disease: second-generation AA group, 562 [24%]; traditional HT group, 876 [8%]; no HT group, 129 [0.7%]). Multivariable Cox proportional hazards analysis showed that the second-generation AA group had an increased risk of depression compared with the no HT group (hazard ratio [HR], 2.15; 95% CI, 1.79-2.59; *P* < .001) and the traditional HT group (HR, 2.26; 95% CI, 1.88-2.73; *P* < .001), including specifically among those with metastatic disease at diagnosis (HR, 2.40; 95% CI, 1.38-4.15; *P* = .002).

**Conclusions and Relevance:**

In this cohort study, patients with prostate cancer who received a second-generation AA had a large and clinically significant increased risk of depression compared with patients who received traditional HT alone or no HT, including when limiting our analysis to individuals with metastatic disease at diagnosis.

## Key Points

**Question:**

Are second-generation antiandrogens (AAs) associated with increased risk of depression among older men diagnosed with prostate cancer?

**Findings:**

In this cohort study of 30 069 men aged 66 years and older, there was a statistically significant 2-fold increase in depression among patients treated with second-generation AA compared with traditional forms of hormone therapy (HT) and no HT.

**Meaning:**

These findings suggest that use of second-generation AAs is associated with a clinically significant increased risk of depression when compared with traditional HT alone or no HT.

## Teaser

This cohort study tests the a priori hypothesis that second-generation AAs are associated with an increased risk of depression, including compared with traditional forms of HT.

## Introduction

Prostate cancer accounts for more than 1 in 5 of all new cancers diagnosed in the United States.^1^ Hormone therapy (HT), including androgen deprivation therapy (ADT), is frequently used in localized prostate cancer along with radiation and is a mainstay of treatment for metastatic, locoregional, and recurrent disease.^2,3^

HT deprives prostate cancer cells of the androgen stimulation that promotes prostate cancer growth and progression, resulting in improvements in overall survival.^4^ HT is typically achieved through medical castration, or androgen deprivation, with luteinizing hormone-releasing hormone (LHRH) agonists with or without antiandrogen (AA) therapy. Despite high initial response rates, nearly all men with advanced prostate cancer progress to castration-resistant disease, which can be treated with second-generation AAs.^5,6^ Second-generation AAs are also used in combination with traditional forms of HT as first-line therapy in patients with hormone-naive metastatic prostate cancer. Second-generation AAs, which work by both inhibiting androgen production (abiraterone) and as androgen receptor antagonists (apalutamide, darolutamide, and enzalutamide), lead to a more profound decrease in androgen signaling than prior therapies.^6^ This increased potency may carry a greater risk of adverse effects related to blocking and suppressing testosterone.^7^

Prior studies have shown a consistent association between ADT and depression using both depression inventory testing and claims data.^8,9^ More recently, depression instrument and patient-reported outcome measures data have emerged that support a potential association of second-generation AAs with symptoms of depression.^7^ Whether there is an association between second-generation AAs and a clinical diagnosis of depression is unknown.

We hypothesized that second-generation AAs would be associated with an increased risk of depression, including compared with traditional forms of HT. This may be particularly clinically relevant, as depression is associated with decreased overall survival in patients with prostate cancer.^10,11^ Considering the prevalence of prostate cancer and the increasing use of second-generation AAs, an association between second-generation AAs and depression may have significant public health implications.

## Methods

### Cohort Selection

**Figure 1.**
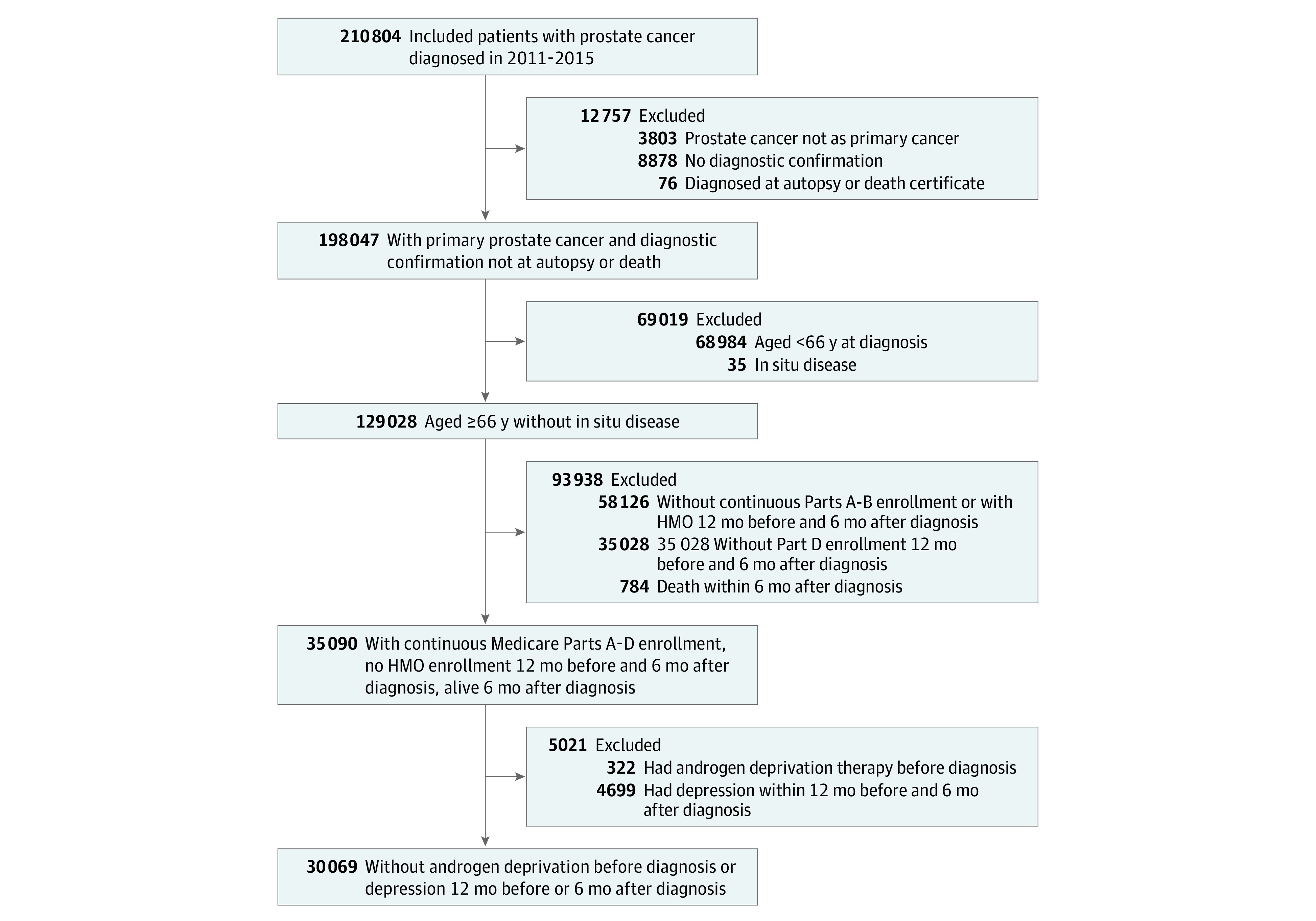
Cohort Selection Flow Diagram HMO indicates health maintenance organization.

We used the National Cancer Institute’s Surveillance, Epidemiology, and End Results (SEER)–Medicare and the Texas Cancer Registry (TCR)–Medicare linked databases. We included men with a first primary prostate cancer diagnosis of localized, regional, or distant stage diagnosed at age 66 years or older from January 2011 to December 2015 without a second cancer within 12 months. We included patients with continuous Medicare Parts A, B, and D coverage, without health maintenance organization enrollment, from 12 months before until 6 months after diagnosis. We excluded individuals who received any form of HT (LHRH agonists/antagonists, AAs) prior to prostate cancer diagnosis, those diagnosed with depression from 12 months before through 6 months after prostate cancer diagnosis, and those who did not survive at least 6 months following prostate cancer diagnosis (Figure 1). The study was deemed exempt from review by the MD Anderson Cancer Center institutional review board and followed Strengthening the Reporting of Observational Studies in Epidemiology (STROBE) reporting guidelines. A waiver of informed consent was obtained because all data were received deidentified.

### Exposures

We evaluated HT use starting at the prostate cancer diagnosis date using both Healthcare Common Procedure Coding System (HCPCS) codes and generic names from Part D prescriptions (eTable 1 in the Supplement). We categorized individuals as having no exposure to any form of HT (no HT group), exposure to HT without any exposure to a second-generation AA (traditional HT group), and any exposure to a second-generation AA regardless of other HT exposure (second-generation AA group). We used a time-varying exposure variable to categorize exposure to HT with individuals remaining in their given group once they met criteria. We stratified HT users by cumulative duration of HT use, defined as 1 to 6, 7 to 12, and more than 12 months.

### Outcome and Variables

The follow-up period was 6 months after prostate cancer diagnosis to the end of Medicare coverage. Patients were censored at date of last enrollment or end of data set follow-up, which was December 31, 2017. The primary outcome was depression, which was defined by the presence of any relevant *International Classification of Diseases, Ninth Revision* (*ICD-9*) or *International Statistical Classification of Diseases and Related Health Problems, Tenth Revision* (*ICD-10*) diagnosis code (eTable 1 in the Supplement) using Medicare claims data, including physician, outpatient, and inpatient claims.^8^ Demographic and clinical variables included year and age of diagnosis, race and ethnicity (as reported by the SEER and TCR registries; race and ethnicity were reported as Black, Hispanic, White, and other [American Indian or Alaska Native, Asian (ie, Chinese, Filipino, and Japanese), Native Hawaiian or Pacific Islander, 2 or more races, and other or unspecified]), marital status, education and poverty quartile, state buy-in (an indication that the beneficiary received Medicaid or other state assistance for those with low income), and residence area as well as T stage, N stage, stage, and grade extracted from the SEER or TCR patient enrollment file. Quartiles of percentage of non–high school education and poverty were estimated based on 2000 census tract. We calculated the Charlson Comorbidity Index score based on physician, inpatient, and outpatient claims in the 12 months before prostate cancer diagnosis.^12^ We defined chemotherapy, radiation, and surgery based on *ICD-9* and *ICD-10* diagnosis and procedure codes and HCPCS codes within 6 months after prostate cancer diagnosis (eTable 1 in the Supplement).

### Statistical Analysis

We compared baseline demographic and clinical variables between groups using χ^2^ tests. We plotted the 1- and 2-year cumulative incidences of depression treating death as a competing risk.^13^ We examined the risk of depression in the second-generation AA, traditional HT, and no HT groups. We calculated the number needed to harm (NNH) to provide a measure of the absolute effect size of second-generation AAs by taking the inverse of the difference between the rates of depression between the no HT group and traditional HT groups compared with the second-generation AA group.

We implemented time-varying exposure multivariable Cox proportional hazards models to determine the association of ADT use with depression using the inverse probability treatment weighted (IPTW) method.^14^ We estimated propensity scores for each group via logistic regression models including year and age of diagnosis, race, marital status, state buy-in, stage, and Charlson comorbidity score. We defined the IPTW as propensity score divided by 1 minus the propensity score for the traditional HT and second-generation AA groups, assigning the no HT group a weight of 1. We checked the postadjustment balance via the standardized difference, with a standardized difference less than 10% indicating good balance. We used a backward selection process and retained the variables in the final multivariable models based on both statistical and clinical significance. Our final model included year of diagnosis, age, race, marital status, state buy-in, stage, Charlson comorbidity score, and surgery within 6 months after diagnosis. We expressed the results in hazard ratios (HRs) or subdistribution hazard ratios (SHRs) and 95% CIs. Subgroup analyses were carried out within localized, regional, and distant disease to address potential confounding by indication. Unweighted analyses were also conducted. The index date for the primary analysis was 6 months after prostate cancer diagnosis (ie, start of exposure).

We conducted a sensitivity analysis using an alternative cohort design in which we defined the start of the follow-up period as 2 years after the diagnosis of prostate cancer, and rather than using a time-varying covariate, exposure groups were defined based only on exposure during that 2-year period. Individuals with a first exposure to HT after the start of the follow-up period were excluded (eTable 2 in the Supplement). The index date for the sensitivity analysis was 2 years after diagnosis of prostate cancer.

We considered *P* < .05 statistically significant, and all tests were 2-tailed. We used SAS version 9.4 (SAS Institute) and R version 4.0.0 (R Project for Statistical Computing).

## Results

Our analytic cohort (Figure 1) included 30 069 patients (11 484 [38%] aged 66-70 years; 22 594 [75%] White) with prostate cancer. Overall, 17 710 (59%) received no HT, 11 311 (38%) received traditional HT only, and 1048 (3%) received a second-generation AA. Baseline characteristics by exposure group (Table 1) were noted to statistically significantly differ for all variables without IPTW. Individuals receiving a second-generation AA were more likely to be older (aged ≥81 years: second-generation AA group, 246 [24%]; traditional HT group, 1997 [18%]; no HT group, 1173 [7%]) and present with advanced disease (eg, distant disease: second-generation AA group, 562 [24%]; traditional HT group, 876 [8%]; no HT group, 129 [0.7%]). After IPTW adjustment, all standardized differences were less than 10% except for year of diagnosis, age, race, and marital status when comparing the no HT and second-generation AA groups, which were approximately 20%. Median follow-up time from diagnosis was 3.8 years (range, 0.5-7 years).

**Table 1.** Baseline Characteristics of Compared Groups

Characteristic	Patients, No. (%)				*P* value
	Total (N = 30 069)	No HT (n = 17 710)	Traditional HT (n = 11 311)	Second-generation AA (n = 1048)	
Year of diagnosis					
2011	6469 (22)	3774 (21)	2466 (2)	229 (22)	<.001
2012	5312 (18)	3091 (18)	1979 (18)	242 (23)	
2013	5651 (19)	3321 (19)	2118 (19)	212 (20)	
2014	6077 (20)	3595 (20)	2247 (20)	235 (22)	
2015	6560 (22)	3929 (22)	2501 (22)	130 (12)	
Age at diagnosis, y					
66-70	11 484 (38)	8134 (46)	3066 (27)	284 (27)	<.001
71-75	9683 (32)	5842 (33)	3564 (32)	277 (26)	
76-80	5486 (18)	2561 (15)	2684 (24)	241 (23)	
≥81	3416 (11)	1173 (7)	1997 (18)	246 (24)	
Race and ethnicity					
Black	2822 (9)	1552 (9)	1185 (11)	85 (8)	<.001
Hispanic^a^	2669 (9)	1422 (8)	1151 (10)	96 (9)	
White	22594 (75)	13660 (77)	8146 (72)	788 (75)	
Other^b^	1984 (7)	1076 (6)	829 (7)	79 (8)	
Marital status					
Married	17 337 (58)	10 498 (59)	6233 (55)	606 (58)	<.001
Single	5034 (17)	2718 (15)	2080 (18)	236 (23)	
Missing	7698 (26)	4494 (25)	2998 (27)	206 (20)	
Education quartile					
1, most education	7686 (26)	4807 (27)	2613 (23)	266 (25)	<.001
2	7056 (24)	4191 (24)	2619 (23)	246 (24)	
3	6811 (23)	3889 (22)	2685 (24)	237 (23)	
4, least education	7378 (25)	3892 (22)	3209 (28)	277 (26)	
Missing	1138 (4)	931 (5)	185 (2)	22 (2)	
Poverty quartile					
Q1, most wealth	7499 (25)	4616 (26)	2618 (23)	265 (25)	<.001
Q2	7279 (24)	4333 (25)	2699 (24)	247 (24)	
Q3	6976 (23)	3962 (22)	2784 (25)	230 (22)	
Q4, least wealth	7177 (24)	3868 (22)	3025 (27)	284 (27)	
Missing	1138 (4)	931 (5)	185 (2)	22 (2)	
State buy-in					
None	25 372 (84)	15 510 (88)	9042 (80)	820 (78)	<.001
Full or partial	4697 (16)	2200 (12)	2269 (20)	228 (22)	
Residence area					
Metropolitan	24 252 (81)	14 199 (80)	9208 (81)	845 (81)	.04
Urban/rural	5817 (19)	3511 (20)	2103 (19)	203 (19)	
T stage					
T1	17 048 (57)	11 166 (63)	5564 (49)	318 (30)	<.001
T2	9740 (32)	5318 (30)	4100 (36)	322 (31)	
T3	1006 (3)	254 (1)	637 (6)	115 (11)	
T4	319 (1)	40 (0.2)	190 (2)	89 (9)	
Missing	1956 (7)	932 (5)	820 (7)	204 (20)	
N stage					
N0	25 627 (85)	15 724 (89)	9293 (82)	610 (58)	<.001
N1	884 (3)	104 (0.6)	554 (5)	226 (22)	
Missing	3558 (12)	1882 (11)	1464 (13)	212 (20)	
Stage					
Localized	23 761 (79)	14 997 (85)	8466 (75)	298 (28)	<.001
Regional	3251 (11)	1766 (10)	1356 (12)	129 (12)	
Distant	1567 (5)	129 (0.7)	876 (8)	562 (54)	
Missing	1490 (5)	818 (5)	613 (5)	59 (6)	
Grade					
Low	14 565 (48)	11 241 (64)	3269 (29)	55 (5)	<.001
High	13 327 (44)	5438 (31)	7113 (63)	776 (74)	
Missing	2177 (7)	1031 (6)	929 (8)	217 (21)	
Charlson comorbidity score					
0	17 470 (58)	10 981 (62)	5904 (52)	585 (56)	<.001
1	6971 (23)	3890 (22)	2850 (25)	231 (22)	
≥2	5628 (19)	2839 (16)	2557 (23)	232 (22)	
Chemotherapy in 6 mo after diagnosis	12 614 (42)	2337 (13)	9354 (83)	923 (88)	<.001
Surgery in 6 mo after diagnosis	6857 (23)	5398 (31)	1348 (12)	111 (11)	<.001
Radiation in 6 mo after diagnosis	24 924 (83)	14 031 (79)	10 160 (90)	733 (70)	<.001

Abbreviations: AA, antiandrogen; HT, hormone therapy.

^a^ Hispanic ethnicity is tabulated independently of race, so Hispanic persons may be of any race.

^b^ Includes American Indian or Alaska Native, Asian (ie, Chinese, Filipino, and Japanese), Native Hawaiian or Pacific Islander, 2 or more races, and other or unspecified.

**Figure 2.**
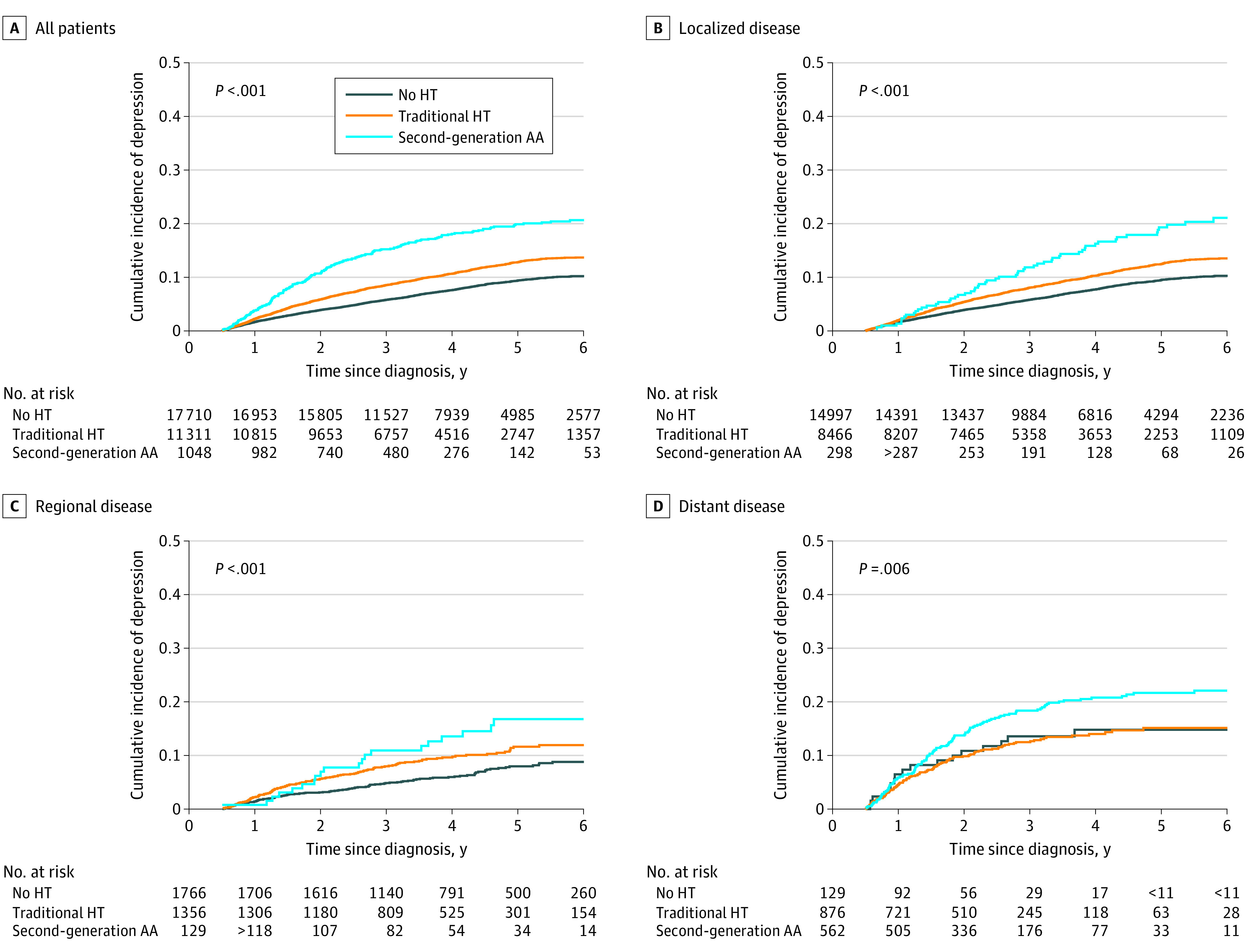
Estimates of the Cumulative Incidence of Depression Since Prostate Cancer Diagnosis by Exposure Group AA indicates antiandrogen; HT, hormone therapy.

The 1- and 2-year cumulative incidence of depression was highest in the second-generation AA group vs the traditional HT and no HT groups, including with stratification by stage (Figure 2 and Table 2). When examining only individuals with metastatic disease at diagnosis, the cumulative 2-year incidence of depression remained highest in the second-generation AA group (17.20%; 95% CI, 14.20%-20.46%) vs the traditional HT (11.49%; 95% CI, 9.46%-13.73%) and no HT groups (12.65%; 95% CI, 7.40%-19.38%).

**Table 2.** Estimate of Cumulative Incidence of Depression via Competing Risks Approach, Starting From 6 Months After Prostate Cancer Diagnosis

Exposure group	Patients, No.		Depression incidence (95% CI)		*P* value
	Total	Event	1-y	2-y	
All	30 069	2764	3.55 (3.34-3.76)	6.03 (5.76-6.31)	NA
By HT use					
No HT	17 710	1344	2.85 (2.61-3.1)	4.79 (4.47-5.12)	<.001
Traditional HT	11 311	1219	4.24 (3.88-4.62)	7.24 (6.77-7.73)	
Second-generation AA	1048	201	7.92 (6.39-9.66)	13.71 (11.7-15.87)	
Localized disease					
No HT	14 997	1150	2.77 (2.51-3.04)	4.79 (4.45-5.15)	<.001
Traditional HT	8466	890	3.8 (3.41-4.23)	6.74 (6.21-7.29)	
Second-generation AA	298	57	5.0 (2.94-7.94)	10.10 (7.0-13.86)	
Regional disease					
No HT	1766	111	2.76 (2.06-3.61)	4.01 (3.15-5.02)	<.001
Traditional HT	1356	128	4.51 (3.50-5.72)	6.70 (5.44-8.13)	
Second-generation AA	129	20	3.88 (1.44-8.27)	8.54 (4.51-14.18)	
Distant disease					
No HT	129	17	9.10 (4.80-15.08)	12.65 (7.40-19.38)	.006
Traditional HT	876	116	7.54 (5.91-9.42)	11.49 (9.46-13.73)	
Second-generation AA	562	117	10.51 (8.14-13.21)	17.20 (14.2-20.46)	

Abbreviations: AA, antiandrogen; HT, hormone therapy; NA, not applicable.

IPTW multivariable adjusted Cox proportional hazards analysis showed that both the traditional HT and second generation AA groups had a statistically significantly increased risk of depression compared with the no HT group (eg, second-generation AA vs no HT: HR, 2.15; 95% CI, 1.79-2.59; *P* < .001) (Table 3). Our results were consistent in the unweighted analysis and when examining individuals with metastatic disease at diagnosis (HR, 2.40; 95% CI, 1.38-4.15; *P* = .002). Additionally, those receiving second-generation AA were significantly more likely to develop depression than those receiving only traditional HT (HR, 2.26; 95% CI, 1.88-2.73; *P* < .001), including with stratification by localized (HR, 2.73; 95% CI, 2.19-3.42; *P* < .001), regional (HR, 3.02; 95% CI, 1.99-4.60; *P* < .001), and distant (HR, 2.47; 95% CI, 1.40-4.36; *P* = .002) disease.

**Table 3.** Multivariable Cox Proportional Hazards Models for the Association of Hormone Therapy With Depression, Using Time-Varying Exposure and Competing Risks Approach Among 30 069 Participants Based on Unweighted and IPTW Cohorts^a^

Exposure group	Unweighted		IPTW	
	SHR (95% CI)	*P* value	SHR (95% CI)	*P* value
All				
No HT	1 [Reference]	NA	1 [Reference]	NA
Traditional HT	1.39 (1.22-1.58)	<.001	1.29 (1.15-1.45)	<.001
Second-generation AA	2.07 (1.68-2.55)	<.001	2.15 (1.79-2.59)	<.001
Localized disease				
No HT	1 [Reference]	NA	1 [Reference]	NA
Traditional HT	1.30 (1.13-1.49)	<.001	1.30 (1.15-1.46)	<.001
Second-generation AA	2.53 (1.80-3.57)	<.001	2.64 (2.12-28)	<.001
Regional disease				
No HT	1 [Reference]	NA	1 [Reference]	NA
Traditional HT	1.22 (0.85-1.76)	.28	1.15 (0.85-1.55)	.37
Second-generation AA	2.16 (1.20-3.88)	.01	2.18 (1.41-3.35)	<.001
Distant disease				
No HT	1 [Reference]	NA	1 [Reference]	NA
Traditional HT	1.17 (0.60-2.29)	.65	0.74 (0.31-1.78)	.51
Second-generation AA	2.51 (1.83-3.44)	.001	2.40 (1.38-4.15)	.002

Abbreviations: AA, antiandrogen; HT, hormone therapy; IPTW, inverse probability treatment weights; NA, not applicable; SHR, subdistribution hazard ratio.

^a^ All analyses in table adjusted for year of diagnosis, age, race, marital status, education and poverty quartile, state buy-in, area of residence, stage, grade, Charlson Comorbidity Index, chemotherapy, radiation, and surgery within 6 months after diagnosis. Models were also adjusted for depression after 6 months post diagnosis but before first use of HT.

The NNH for the occurrence of depression was 9 for the use of any second-generation AA compared with no HT and 12 when compared with traditional HT only. Among individuals with metastatic disease at diagnosis, the NNH was 13 for the use of any second-generation AA compared with no HT and 13 compared with traditional HT only.

We conducted a sensitivity analysis using an alternative study design (eTable 2 in the Supplement) in which groups were assigned based on HT exposure in the 2 years following prostate cancer diagnosis and follow-up for depression diagnoses started at that 2-year point (eTable 3 in the Supplement). Using this approach, we found consistent results to our primary analysis, with exposure to a second-generation AA being statistically significantly associated with depression in the full cohort (HR, 2.68; 95% CI, 1.86-3.87; *P* < .001). Our results were not statistically significant when examining individuals with metastatic disease at diagnosis (HR, 1.15; 95% CI, 0.23-5.78; *P* = .86). Finally, we found a statistically significant association between depression and decreased overall survival in all 3 groups (ie, no HT, traditional HT, and second-generation AA) (eTable 4 in the Supplement).

## Discussion

In this large retrospective cohort study, patients with prostate cancer who received a second-generation AA had a clinically relevant and statistically significant absolute increased risk of depression compared with patients who received traditional HT alone or never received HT. Importantly, our results were consistent when examining only individuals with distant disease at diagnosis. This association remained statistically significant after adjusting for extensive demographic and clinical variables and when applying propensity score–based weighting.

Depression rates are high among patients with cancer,^15^ and depression is associated with mortality in patients with cancer and specifically among men with prostate cancer.^10,11^ In our analysis, we found that across all HT exposure groups, a post–prostate cancer diagnosis of depression was associated with worse overall survival. While past studies have supported an association between ADT and a diagnosis of depression,^8,16,17,18^ to our knowledge our data are the first to find higher rates of depression diagnoses with a second-generation AA.

Second-generation AAs have been shown to significantly increase survival when compared with no HT and primary ADT alone, especially in patients with metastatic prostate cancer.^19,20^ Second-generation AAs have been proven to be beneficial in the treatment of prostate cancer, most importantly due to their ability to prolong life in patients with late-stage disease who have fewer safe and effective treatment options. However, their association with depression should not be overlooked, particularly considering that depression can be identified and treated, allowing these patients to improve not only their quantity of life but also its quality.

Interventions to reduce depression have been shown to increase survival in both patients with cancer and older individuals without cancer.^21,22^ For example, there is evidence that cognitive behavioral stress management interventions in patients with coronary heart disease are effective in improving clinical course and can reduce overall mortality.^23,24^ While some approaches to mitigate the cognitive consequences of ADT have been identified,^25^ there is a paucity of validated evidence on traditional depression treatments in patients with cancer.^26^ As second-generation AA therapy is now being increasingly prescribed across different disease states of prostate cancer (including hormone-naive metastatic disease, M0 castrate-resistant disease, and metastatic castrate-resistant disease),^6,27^ our study suggests that prospective research is needed to determine the effect of depression on prostate cancer clinical outcomes and the ability of interventions to prevent, identify, and treat depression in this patient group.

The etiology of depression in patients receiving second-generation AA therapy is likely multifactorial. Androgen receptors are widespread in the central nervous system and have been shown to have a role in cognition and anxiety.^28,29^ There is evidence that central testosterone signaling has neuroprotective effects.^30^ While the association between endogenous hormones and depressive disorders is complex, low testosterone may be causally related to depression. Decreased global testosterone levels have been shown to cause depressive symptoms and decreased mood,^31^ including in previously healthy men.^32,33,34^ Furthermore, increasing testosterone concentrations have been shown to be beneficial with respect to mood and depressive symptoms among symptomatic men with hypoandrogenism.^35^ This might be partially due to the role of testosterone in regulation of other key neurochemicals related to mood, such as serotonin, norepinephrine, and dopamine.^36^ It is possible that hormone therapy may indirectly increase the risk of depression through a decrease in the overall quality of life due to poor physical health and numerous testosterone-related side effects, which include neurocognitive dysfunction, hot flushes, fatigue, gynecomastia, insomnia, libido loss, sexual dysfunction, and osteoporosis.^8,37,38,39,40^

Most of the evidence supporting the association between HT and depression is limited to studies of LHRH agonists. Despite the growing role of second-generation AAs in the treatment of prostate cancer, however, the association between these novel agents and depression has not been well studied. A recent systematic review examined existing studies investigating the association of second-generation AAs with depression.^7^ The authors found that no prior studies have examined the association of second-generation AAs with a clinical diagnosis of depression. Prior studies were limited to those examining the association of second-generation AAs with patient well-being questionnaires and research-based depression inventories such as the Functional Assessment Cancer Therapy–Prostate emotional well-being subscale. Interestingly, these studies showed evidence of improved emotional well-being with receipt of second-generation AAs compared with prednisone or placebo,^41,42,43,44^ which the authors attributed to potential improvement in disease control. There was no clear difference in emotional well-being when second-generation AAs were compared with first-generation AAs, but the existing data were limited by small sample sizes.^45^ Ultimately, nondiagnostic patient-reported depressive symptomology and instrument data cannot substitute for clinical diagnostic outcome measures,^46^ as examined in the current study.

### Limitations

Our study has limitations. First, our SEER-Medicare cohort was limited to men aged 66 years and older at diagnosis. While our results may not be generalizable to younger men, most patients diagnosed with prostate cancer are older.^1^ Second, the design of our study is retrospective; thus, we are unable to fully assess causality. Third, our study is limited by the nature of large claims data, which rely on the accuracy of diagnostic codes and are not comprehensive of all patient characteristics. Notably, administrative claims data have been shown to underestimate the true incidence of depression.^47^ Fourth, the adjustment for patient characteristics based on Charlson Comorbidity Index was done prior to the diagnosis of prostate cancer. Therefore, it is possible that it does not reflect the burden of comorbidity at the time of treatment with second-generation AAs. Fifth, our second-generation AA group mostly included men who were also exposed to traditional forms of HT and therefore had a longer cumulative exposure to HT of any form. However, we did not observe a duration-dependent increase in depression risk with exposure to traditional HT in our full cohort, and therefore, a longer total duration of HT exposure does not appear to completely explain the magnitude of increased risk of depression observed in the second-generation AA group. Sixth, we excluded individuals with prevalent depression from our study given the limitations of examining the worsening of existing depression in claims-based analyses. However, the outcomes associated with second-generation AAs among men who carry a pre–AA therapy diagnosis of depression is critical and should be examined in future studies. Seventh, men receiving second-generation AAs may be more likely to receive care at research and academic centers and therefore may have more access to ancillary services that would increase their likelihood of being diagnosed with depression. However, we were unable to account for this in our analysis.

Furthermore, men who are receiving a second-generation AA may be more likely to have advanced disease and be receiving second-line therapies after the failure of first-line treatments. Therefore, the second-generation AA group may have been more likely to experience depression secondary to prostate cancer severity and an adverse treatment course. Our results were consistent when examining only individuals with distant disease at diagnosis in our primary analysis. In our sensitivity analysis, in which we defined the start of the follow-up period as 2 years after the diagnosis of prostate cancer, we did not observe a statistically significantly increased risk when limiting our analysis to individuals with metastatic disease at diagnosis. However, it is difficult to interpret this finding given the limited power of this secondary subgroup analysis. Importantly, as our data set does not capture clinical progression (eg, transition to castrate-resistant disease and second-line therapy), our analysis does not fully account for confounding by indication (ie, individuals more likely to both have depression and more likely to receive second-generation AAs because of their disease course). Our results should be validated in other data sets that, ideally, contain information on patient and disease characteristics at the time of receipt of HT.

## Conclusions

In our cohort of more than 30 000 US men aged 66 years with prostate cancer, we observed a clinically relevant and statistically significant association between the incidence of depression in men taking second-generation AAs when compared with men receiving traditional forms of HT or men not taking HT. While our results were consistent when examining only individuals with metastatic disease at diagnosis, our results should be validated in future studies designed to account for second-generation AA indication (eg, castration resistance). Considering that recipients of second-generation AAs have regular health care exposure due to the nature of their treatment, early depression screening and treatment are feasible interventions that could greatly improve their quality of life and clinical outcomes. The possible increased risk of depression with second-generation AA use should be discussed with patients, and depression screening should be considered in all recipients of HT, in particular those who receive second-generation AA therapies.

## Notes

Supplement. **eTable 1.** Codes and Medication Names Used to Identify Diagnosis and Treatment **eTable 2.** Alternative Cohort Selection Criteria **eTable 3.** Multivariable Cox Proportional Hazards Model for the Association of Hormone Therapy Use Within 2 Years Post Diagnosis With Depression After 2 Years Post Diagnosis via Competing Risks Approach for the Unweighted and IPTW Cohorts **eTable 4.** Multivariable Cox Proportional Hazards Model for the Association of Depression (Yes vs No) and Overall Survival
