# Supplementary material for: Association of Second-generation Antiandrogens With Depression Among Patients With Prostate Cancer
Source: JAMA Netw Open. 2021 Dec 23;4(12):e2140803. doi: 10.1001/jamanetworkopen.2021.40803 (PMC8703250; doi:10.1001/jamanetworkopen.2021.40803)
Supplement: Supplement. — eTable 1. Codes and Medication Names Used to Identify Diagnosis and Treatment eTable 2. Alternative Cohort Selection Criteria eTable 3. Multivariable Cox Proportional Hazards Model for the Association of Hormone Therapy Use Within 2 Years Post Diagnosis With Depression After 2 Years Post Diagnosis via Competing Risks Approach for the Unweighted and IPTW Cohorts eTable 4. Multivariable Cox Proportional Hazards Model for the Association of Depression (Yes vs No) and Overall Survival [file jamanetwopen-e2140803-s001.pdf]

Supplemental Online Content

Nowakowska MK, Lei X, Wehner MR, Corn PG, Giordano SH, Nead KT. Association of second-generation antiandrogens with depression among patients with prostate cancer. *JAMA Netw Open.* 2021;4(12):e2140803. doi:10.1001/jamanetworkopen.2021.40803

eTable 1. Codes and Medication Names Used to Identify Diagnosis and Treatment

eTable 2. Alternative Cohort Selection Criteria

eTable 3. Multivariable Cox Proportional Hazards Model for the Association of Hormone Therapy Use Within 2 Years Post Diagnosis With Depression After 2 Years Post Diagnosis via Competing Risks Approach for the Unweighted and IPTW Cohorts

eTable 4. Multivariable Cox Proportional Hazards Model for the Association of Depression (Yes vs No) and Overall Survival

This supplemental material has been provided by the authors to give readers additional information about their work.

© 2021 Nowakowska MK et al. *JAMA Network Open.*

eTable 1. Codes and Medication Names Used to Identify Diagnosis and Treatment

	Diagnosis code	Procedure code	HCPCS/CPT code
**Diagnosis**			
Depression	(ICD-9) 296.2, 296.3, 296.5, 296.6, 298.0, 300.4, 301.10, 301.12, 301.13, 309.0, 309.1, 311 (ICD-10) F31.3, F31.4, F31.5, F31.6, F32, F33, F34.0, F34.1, F38.1, F43.21		
**Treatment**			
Hormone therapy			J0128, J1675, J1950, J3315, J9155, J9202, J9217, J9218, J9219, J9225, J9226, C9216, C9430, S0133, S0165, Q2020
Orchiectomy		(ICD-9) 62.3, 62.4, 62.41, 62.42 (ICD-10) 0VTC0ZZ, 0VTC4ZZ, 0VT90ZZ, 0VT94ZZ, 0VTB0ZZ, 0VTB4ZZ	54520, 54521, 54522, 54530, 54535,
Surgery Radical prostatectomy Laparoscopic (radical prostatectomy, Robotic-assisted laparoscopic radical prostatectomy)		17.42, 40.1, 40.2, 40.3, 40.5, 40.53, 40.59, 60.21, 60.29, 60.2–60.6, 60.51-60.59, 60.61, 60.62, 60.69	54690, 55810, 55812, 55815, 55821, 55831, 55840, 55842, 55845, 55866, 55899, S2900

© 2021 Nowakowska MK et al. *JAMA Network Open.*

Radiation: EBRT	V58.0,<br>V66.1, V67.1	60.99, 92.2,<br>92.20,<br>92.21-92.29,<br>92.3,<br>92.30-92.39,<br>92.4,<br>92.41	0073T, 0082T, 0182T, 0197T,<br>61793, 76872, 76873, 76965, 77261–77799, C1715, C1717, C2638-C2641, C2698, C2699, G0173, G0251, G0256, G0261, G0339, G0340, G0458, G6003, G6005, G6006, G6015, Q3001, S8049;<br>Revenue center codes 0330 or 0333, 0339 or 0342
Radiation: brachythera py			0182T, 77750-77799, C1715, C1716, C1717, C1719, C2616, C2634-C2645, C2698, C2699, G0458, Q3001
Chemotherapy	V58.1<br>V66.2, V67.2	99.25	95990, 95991, 96400-96549,<br>96530, J0640, J2405, J8520 - J9999 K0415, K0416, Q0083-Q0085, Q0179, S0177, S0181<br>Revenue center codes 0331, 0332, or 0335;<br>For 2005 only, use these G0355-G0363, G9021-G9032

**Description**	**Generic Names**	**HCPCS Codes**
ADT	(GnRH agonist) Leuprolide, Groserelin, Triptorelin, Histrelin<br>(GnRH antagonist) Degarelix, Abarelix<br>(Anti-androgen) Bicalutamide, Nilutamide, Flutamide<br>(CYP17 inhibitors) Abiraterone	J0128, J1675, J1950, J3315, J9155, J9202, J9217, J9218, J9219, J9225, J9226,<br>C9216, C9430, S0133, S0165, Q2020

© 2021 Nowakowska MK et al. *JAMA Network Open.*

	Enzalutamide, Apalutamide, Darolutamide	

ADT, androgen deprivation therapy; GnRH, gonadotropin-releasing hormone.

C9216 Injection, abarelix for injectable suspension, per 10 mg

C9430 Leuprolide acetate, per 1 mg, brand name

J0128 Injection, abarelix for injectable suspension, per 10 mg

J1675 Injection, histrelin acetate, 10 mcg

J1950 Injection, leuprolide acetate (for depot suspension), per 3.75 mg

J3315 Injection, triptorelin pamoate, 3.75 mg

J9155 Injection, degarelix, 1 mg

J9202 Goserelin acetate implant, per 3.6 mg

J9217 Leuprolide acetate (for depot suspension), 7.5 mg

J9218 Leuprolide acetate, per 1 mg

J9219 Leuprolide acetate implant, 65 mg

J9225 Histrelin implant (Vantas), 50 mg

J9226 Histrelin implant (Supprelin LA), 50 mg

S0133 Histrelin, implant, 50 mg

S0165 Injection, abarelix, 100 mg

Q2020 Injection, histrelin acetate, 10 mcg

© 2021 Nowakowska MK et al. *JAMA Network Open.*

eTable 2. Alternative Cohort Selection Criteria

(Coverage within 1 year before and 2 years after cancer diagnosis, hormone therapy was measured within 2 years after cancer diagnosis).

Step	Cohort Selection Criteria	N
1	Prostate cancer diagnosed SEER/TCR 2011- 2015.	210,804
2	First primary prostate cancer.	207,001
3	Diagnostic confirmation.	198,123
4	Diagnosed not at autopsy or death certificate.	198,047
5	Age 66+ at diagnosis.	129,063
6	Excluded patients with *in-situ* diseases.	129,028
7	Medicare Parts A & B coverage and no HMO within 1 year before and 2 years after prostate cancer diagnosis.	67,384
8	Part D coverage within 1 year before and 2 years after prostate cancer diagnosis.	26,145
9	Excluded patients who died within 2 years post prostate cancer diagnosis.	23,834
10	Excluded patients who had hormone therapy within 1 year before or initiated hormone therapy after 2 years post prostate cancer diagnosis.	23,107
11	Excluded patients who had depression from within 1 year before and 2 years after prostate cancer diagnosis.	19,296

© 2021 Nowakowska MK et al. *JAMA Network Open.*

eTable 3. Multivariable Cox Proportional Hazards Model for the Association of Hormone Therapy Use Within 2 Years Post Diagnosis With Depression After 2 Years Post Diagnosis via Competing Risks Approach for the Unweighted and IPTW Cohorts

	Unweighted Cohort			IPTW Cohort		
**All patients (N=19296)**	**HR**	**95% CI**	**P-value**	**SHR**	**95% CI**	**P-value**
Traditional-HT v. no-HT	1.24	1.03-1.49	0.02	1.17	0.99-1.37	0.06
Second-generation AA v. no-HT	2.61	1.71-3.99	<0.001	2.68	1.86-3.87	<0.001
**Localized diseases (N=15801)**						
Traditional-HT v. no-HT	1.26	1.03-1.54	0.027	1.20	1.00-1.43	0.05
Second-generation AA v. no-HT	3.73	1.88-7.42	<0.001	3.58	2.28-5.62	<0.001
**Regional diseases (N=2027)**						
Traditional-HT v. no-HT	1.10	0.61-1.97	0.75	0.98	0.59-1.63	0.94
Second-generation AA v. no-HT	2.72	0.74-10.03	0.13	2.65	0.99-7.13	0.05
**Distant diseases (N=617)**						
Traditional-HT v. no-HT	0.63	0.17-2.36	0.50	0.61	0.14-2.65	0.51
Second-generation AA v. no-HT	1.22	0.30-4.97	0.78	1.15	0.23-5.78	0.86

AA, antiandrogens; CI, confidence interval; HR, hazard ratio; HT, hormone therapy; IPTW, inverse probability treatment weights; SHR, subdistribution hazard ratio; v, versus. Adjusted for year of diagnosis, age, race, marital status, education and income quartile, state buy-in, residence area, stage, grade, Charlson comorbidity index excluding dementia, chemotherapy, radiation, and surgery within 12-months of diagnosis.

© 2021 Nowakowska MK et al. *JAMA Network Open.*

eTable 4. Multivariable Cox Proportional Hazards Model for the Association of Depression (Yes vs No) and Overall Survival

HT group	Stage	N	HR	95% CI	P-value
**No-HT**	All stage	17710	1.96	1.72-2.23	<0.001
	Localized	14997	2.07	1.79-2.38	<0.001
	Regional	1766	3.55	2.13-5.93	<0.001
	Distant	129	0.92	0.36-2.34	0.86
**Traditional-HT**	All stage	11311	1.34	1.20-1.50	<0.001
	Localized	8466	1.48	1.30-1.70	<0.001
	Regional	1356	1.74	1.17-2.59	0.007
	Distant	876	0.93	0.72-1.21	0.60
**Second-generation AA**	All stage	1048	1.28	1.07-1.53	0.006
	Localized	298	1.55	1.09-2.21	0.02
	Regional	129	1.77	0.93-3.38	0.08
	Distant	562	1.11	0.88-1.41	0.38

AA, antiandrogens; CI, confidence interval; HR, hazard ratio; HT, hormone therapy; HR, hazard ratio; v, versus. Adjusted for year of diagnosis, age, race, marital status, education and income quartile, state buy-in, residence area, grade, Charlson comorbidity index, chemotherapy, surgery, and radiation within 6-months of diagnosis.

© 2021 Nowakowska MK et al. *JAMA Network Open.*
